# Staff Wellbeing and Engagement: A Strategic Priority at a Hospital in Singapore

**DOI:** 10.3390/healthcare14030391

**Published:** 2026-02-04

**Authors:** Dorcas Yuen Mei Won, Jolene Wei Ling Ooi, Zhen Wei Lew, Sandra En Ting Tan, Soon Noi Goh

**Affiliations:** Changi General Hospital, Singapore 529889, Singapore; jolene.ooi.w.l@singhealth.com.sg (J.W.L.O.); zhen_wei_lew@cgh.com.sg (Z.W.L.); sandra_tan@cgh.com.sg (S.E.T.T.); soon_noi_goh@cgh.com.sg (S.N.G.)

**Keywords:** allied health professionals, staff wellbeing and engagement, joy at work, empowerment, leadership, institutional commitment

## Abstract

**Background:** In today’s dynamic healthcare environment, Changi General Hospital (CGH) has positioned staff wellbeing and engagement as fundamental priorities that underpin workforce sustainability and quality care delivery. Recognizing that allied health professionals (AHPs) face unique emotional demands and potential empathy fatigue, the CGH Allied Health Division (AHD) uses three strategic pillars: individual empowerment, leaders as key stewards and institutional support systems to address staff wellbeing and engagement. This paper will evaluate the outcomes of implementing the programs and identifying the barriers and enablers to achieving staff wellbeing and engagement. **Methods:** It adopts a mixed-methods approach using both quantitative survey data and qualitative feedback. **Results:** A total of 314 AHPs participated with a mean employment duration of 8.89 years. While 95% agreed that their work was meaningful and 76.8% reported happiness at work, 40.8% did not experience being recognized by the organization and approximately 30% did not find higher management responsive to their needs or transparent in their communication. Qualitative analysis revealed concerns about psychological safety of sharing one’s opinions and concerns, and a desire for better renumeration and career progression. **Conclusions:** AHPs reported happiness and meaningfulness in their clinical work. However, issues with organizational recognition, higher management responsiveness and transparency, as well as psychological safety were elicited. Working towards addressing fostering psychological safety and enhancing recognition and communication with management are important in order to develop and sustain a thriving healthcare workforce capable of high-quality patient care. Overall, the findings reinforced AHD direction of putting employee wellbeing and engagement as a strategic priority.

## 1. Introduction

Healthcare systems worldwide increasingly recognize staff wellbeing and engagement as fundamental pillars of high-quality, sustainable healthcare delivery. When healthcare professionals are well-supported and engaged, they demonstrate enhanced clinical performance, improved patient satisfaction, reduced medical errors, and greater innovation in care delivery [1]. This recognition has elevated staff wellbeing from a desirable organizational goal to a strategic imperative that directly impacts patient safety, care quality, and healthcare system resilience. Despite positive developments in healthcare wellness initiatives, significant challenges persist. Systematic reviews indicate that burnout affects healthcare workers globally, with rates varying by specialty, setting, period (pandemic/non-pandemic) and geographic location [2,3]. Burnout, characterized by emotional exhaustion, depersonalization, and reduced personal accomplishment, represents a syndrome of chronic workplace stress that has not been successfully managed [4].

The COVID-19 outbreak further exacerbates the mental health burden, highlighting the need for enhancing mental health support for healthcare professionals. The literature has reported factors associated with personal-, work-, and patient-related burnout among healthcare workers during COVID-19 management, having underlying medical illness, and receiving inadequate psychological support in the workplace [5]. A systematic review showed that one third of healthcare professionals in Asia suffered from depression, anxiety and stress and more than two thirds of them suffered from fear and burnout during the COVID-19 pandemic [6]. A later study found allied health professionals (AHPs) reported the highest rates of emotional exhaustion among all healthcare worker categories and ranked second in depersonalization [7].

At the national level, the Ministry of Manpower (MOM) together with the labor union and the employer federation launched the Tripartite Advisory on Mental Health and Well-being at Workplaces in 2020, which offers practical guidance to employers and highlights available support measures. The Workplace Safety and Health Council also launched a Well-being Champions Network that allows companies to exchange best practices with each other, as well as offers access to resources and training to strengthen workplace mental wellbeing [8]. Singaporean firms are increasingly implementing measures like providing confidential counseling through Employee Assistance Programs (EAPs), training supervisors to recognize and respond to distress and fostering a supportive work environment. Companies are also conducting regular assessments of mental wellbeing using tools like iWorkHealth, reviewing HR policies to promote flexibility and work–life harmony, and hiring individuals with mental health conditions to build more inclusive workplaces.

## 2. Context and Objectives

### 2.1. Study Context and Gaps

SingHealth (a healthcare cluster of which Changi General Hospital, CGH, is a member institution) adopted the Institute for Healthcare Improvement’s Framework with its Joy at Work in 2019 [9]. The adoption was grounded in the fundamental belief that joy at work is essential for delivering exceptional patient care, achieving excellence in practice, and building sustainable careers in healthcare. The Institute for Healthcare Improvement’s Framework on Joy in Work provides a particularly comprehensive model, emphasizing the creation of vital conditions including meaningful work, supportive relationships, psychological safety, and system-wide appreciation for whole-person wellbeing [10]. This framework recognizes that joy at work is not merely the absence of distress, but the presence of positive conditions that enable healthcare professionals to find meaning and satisfaction in their roles. Studies reveal that for effective interventions, healthcare worker wellbeing requires comprehensive, multi-level approaches that address individual, team, and organizational factors simultaneously [11,12]. Individual-focused interventions including mindfulness training and resilience building show promise but achieve optimal impact when complemented by systemic changes [13]. Team-based interventions such as structured debriefing and peer support programs enhance social support and collective resilience [14].

To operationalize this commitment system-wide, SingHealth established a comprehensive Joy at Work workgroup spanning all institutions, with strategic appointment of Joy Enablers and departmental representatives as the Joy Ambassadors creating a network of champions dedicated to fostering positive work environments and translating the framework into practical interventions.

This led to the establishment of the SingHealth Staff Wellness office in April 2022 to bring together representatives from each member institution to implement strategies in five key areas—a psychological safety culture that values open and honest dialog and feedback; career wellness through a supportive work environment; cluster-wide community and people engagement to equip staff with knowledge and tools for holistic wellness; mental wellness support at multi-level and physical wellness initiatives such as health screening and preventive care [15].

### 2.2. Objectives

While the Institute for Healthcare Improvement’s Framework on Joy in Work provides theoretical direction, limited evidence exists regarding its practical implementation in Singapore’s healthcare context, particularly among allied health professionals. This study aims to investigate the workplace wellbeing and engagement experiences among allied health professionals, as well as evaluate this early phase implementation and outcomes of a systematic staff wellbeing intervention adapted from the IHI Joy in Work Framework in a Singapore acute care setting. It will contribute empirical evidence and future development of culturally responsive workplace wellness interventions in an Asian healthcare context.

### 2.3. Study Questions

The primary study question is “What are the workplace wellbeing and engagement experiences of allied health professionals, and the implementation barriers and enablers, as well as the outcomes of this systematic wellbeing intervention based on the Joy at Work Framework?”

Specific study questions:

1. How do the allied health professionals perceive their workplace wellbeing and engagement experiences, particularly for happiness at work, meaningfulness, psychological safety, recognition, and management support?
2. What are the key implementation processes, barriers, and achievements when implementing this systematic wellbeing framework?
3. What are factors influencing the effectiveness of these systematic staff wellbeing interventions in an allied health setting?
4. What are the practical insights and lessons learnt from the implementation of the Joy at Work framework for allied health professionals in Asian healthcare contexts?

## 3. Methods

### 3.1. Study Design

This organizational case study adopted a mixed-methods approach using both quantitative survey data and qualitative feedback simultaneously to provide comprehensive analysis of staff wellbeing and engagement initiatives implemented at CGH AHD.

This paper will provide a qualitative descriptive and reflective account of how CGH AHD framed the Model and implemented the initiatives that drive the Division in staff wellbeing and engagement. It synthesizes and critically analyses the nascent literature on staff wellbeing and engagement, secondary data on the health cluster and organizational level influences, and empirical findings from the design and implementation of the programs.

On the quantitative side, this study leverages a self-designed Staff Wellbeing and Engagement Survey created and conducted at the Division level. It was intended to enable the Division to track staff wellbeing and engagement annually. No demographics except the department to which the staff belonged and the number of years worked were collected. The survey comprised 13 questions designed via the face validity approach by the Wellbeing and Engagement Team with the purpose to directly elicit staff responses of the different aspects of work experience (e.g., happiness at work, meaningfulness, feeling valued, management support, psychological safety) that contribute to their sense of wellbeing and engagement. These aspects captured were based on the practical and contextualized insights of the team and ensured the instrument with enough cultural relevance to the Singapore healthcare context. Conceptualizing staff wellbeing and engagement as involving several aspects is also performed by other organizations. For example, the MAYO clinic All-Staff survey similarly has items asking about job satisfaction and meaning, sense of connection and belonging, fairness in work, communication experience with management and sense of safety speaking up [16]. In the current survey, respondents anonymously rated their work experiences on a four-point Likert scale. The aspects of work experience covered by the survey questions are found in the second table in Section 4.

Qualitatively, there were two qualitative questions eliciting reasons for a respondent’s rating of level of comfort sharing opinions and concerns, and of how one would like to be recognized for one’s work. The questionnaire was sent to all CGH AHPs possessing corporate email accounts via invitation links between July and August 2024. The quantitative responses were analyzed using descriptive statistics (e.g., means and frequencies) using IBM SPSS V28.0.1. The qualitative responses were thematically coded and categorized into major themes. Using Braun and Clarke’s approach, a Team member read and re-read the responses to generate the codes organically from the data [17]. He then grouped the related codes together and clustered into themes. Example codes like “bonus” and “salary increase” are clustered into a theme like monetary rewards. The rest of the Team reviewed and confirmed the narrative constructed with the quantitative data.

### 3.2. Conceptual Framework

At CGH, a tertiary academic hospital in Singapore with a workforce of over 6000 wellness is conceptualized as a multi-dimensional continuum. It spans mental, physical, emotional, social, and occupational domains, acknowledging that the ability to thrive in healthcare requires comprehensive and continuous support across all levels of the organization. CGH implements a structured, multi-pronged wellness framework built on three synergistic pillars: (i) empowering individuals to take proactive ownership of their wellbeing, (ii) strengthening leadership capabilities to cultivate psychologically safe and inclusive team environments, and (iii) embedding systemic and institutional structures that prioritize and sustain wellness. Together, these approaches form an integrated strategy to enhance resilience, engagement, and joy at work.

The first pillar places emphasis on individual empowerment. According to Social Cognitive Theory, one’s capability to exercise some measure of control over adverse events is protective, enabling, and central to post-traumatic recovery [18]. Prati, Pietrantoni and Cicognani [19] found a significant relationship between stress appraisal and professional quality of life of rescue workers among those with low levels of self-efficacy. Self-efficacy buffers the impact of perceived stressful encounters on professional quality of life. Recognizing that personal ownership is a cornerstone of wellbeing, CGH offers accessible, evidence-informed tools and resources to support emotional regulation, stress management, and self-care. Digital campaigns like Project Check-in deliver bite-sized education on sleep, gratitude, and emotional health, while Discovering Our Glimmers workshops encourage staff to reflect moments of joy in daily work. The ReXilience^®^ Programme, available in multiple tiers, offers experiential learning and digital toolkits to support self-awareness and personal resilience. In leadership development, supervisors are trained to facilitate psychologically safe debriefs using frameworks such as the Post-Incident Dialog with Empathy. These initiatives seek to normalize conversations around wellbeing, mental and physical health, reduce stigma, and equip staff with the skills to navigate personal and professional stressors effectively. By fostering emotional literacy and reflective practice, CGH attempts to promote a culture in which wellness becomes both an individual’s responsibility and a shared organizational value.

The second pillar focuses on enabling leaders to serve not only as operational managers but as key stewards of staff wellbeing. Collett, Emad and Gupta‘s study of four hundred healthcare workers in the United Kingdom showed an inverse association between perceived level of managerial or collegial support with depression, anxiety, insomnia and burnout among these staff [20]. Leaders and supervisors are uniquely positioned to influence psychological safety, interpersonal dynamics, and workplace culture. As such, CGH invests in leadership development that nurtures emotional intelligence, compassionate communication, and responsive supervision. Through targeted programs such as tiered resilience training, peer support facilitation, and cross-generational dialog, the institution cultivates leaders who are equipped to engage staff authentically, address distress, and inspire a sense of meaning and belonging in the workplace. Structured mentorship, 360-degree feedback, and forums like Insight Hour further promote inclusive leadership and cross-team connection.

The third pillar reflects CGH’s institutional commitment to embed wellness into the organizational fabric. It echoes the international, national, and healthcare cluster call and action on a systemic approach. By integrating wellbeing into governance, policy, and infrastructure, CGH ensures that wellness is not siloed or incidental, but central to how the institution operates. This includes investment in physical spaces, for example, reCGHarge + Lounge for rest and recovery, access to peer responder networks, confidential counseling and psychological support, dedicated staff clinics, and regular engagement activities. System-wide initiatives such as wellness campaigns and staff recognition programs reinforce a culture of appreciation, trust, and civility. These efforts are strengthened by ongoing evaluation and feedback mechanisms, enabling the institution to adapt and improve its wellness offerings in alignment with evolving staff needs.

### 3.3. Setting and Context

**Figure 1:**
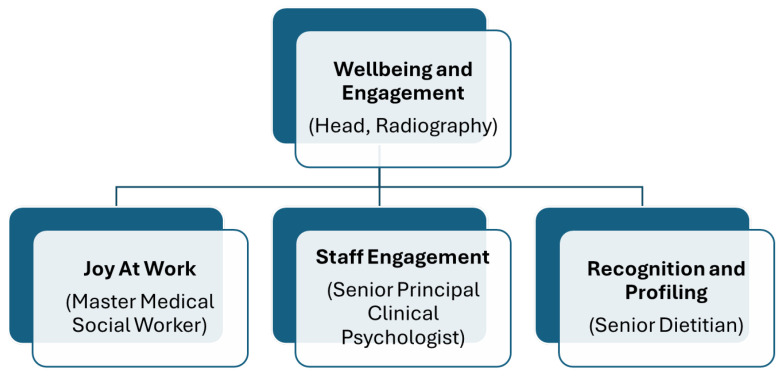
CGH Allied Health Division Wellbeing and Engagement organization structure.

The Division consisted of 715 professionals (in October 2024) representing 23 professional groups, for example, audiologists, clinical psychologists, dietitians, medical social workers, radiographers, and rehabilitation therapists of varying staff strengths. The CGH AHD Wellbeing and Engagement Team was established in April 2024. The Team operates CGH’s three-pillar wellness framework through its own logic framework Joy at Work programs, Staff Profiling and Recognition initiatives, and Staff Engagement strategies to foster resilience, engagement, and joy at work in the Division (see Figure 1). The Wellbeing and Engagement Team is an innovative collaborative model where three domain leads from diverse AHP groups work synergistically to enhance staff wellness, resilience, and sustained joy at work among the AHPs. The Team is headed by the AHD Shared Service Lead in Wellbeing and Engagement whose appointment serves as the strategic coordinator, providing overarching direction and ensuring alignment among the three domains whilst maintaining focus on the broader organizational wellness objectives.

The Joy at Work Lead focuses on creating positive work environments that enhance staff wellbeing and satisfaction through strategic program design, implementation, and impact measurement. This role encompasses fostering meaning and purpose in work, eliminating non-value-added processes, promoting workplace camaraderie, and championing staff wellness initiatives to address burnout and build resilience.

The Staff Engagement Lead specializes in designing and implementing comprehensive engagement strategies that motivate, enable, and energize staff across all organizational levels. This role involves creating structured methodologies to understand staff needs and concerns, analyzing Employee Engagement Survey findings, and fostering psychologically safe environments where staff feel heard and valued.

The Staff Profiling and Recognition Lead establishes strategic recognition frameworks that actively celebrate staff achievements whilst raising awareness of allied health professionals’ roles and contributions. This role encompasses promoting appreciation cultures, consolidating recognition platforms, and ensuring strategic profiling opportunities across various AH professions. For example, the hosting of the International AHP Day Open House, the creation of an e-brochure profiling all Allied Health professions, job observation collaborations with secondary schools, and the CGH Townhall Showcase elevated the Division visibility both within the hospital and publicly. Systematic recognition programs including the Vanguard Award and Galaxy of Stars Award program institutionalized appreciation of the staff.

### 3.4. Key Activities

#### 3.4.1. Empowering Individual Ownership of Well-Being

Two Joyful Engagement sessions with Joy Ambassadors (departmental representatives) in April and August 2024 were conducted to equip staff with tools for personal wellness ownership. Six other activities engaged a total of 246 participants in building personal resilience and purpose. Department-level initiatives further supported individual departments and sections through a further 97 joyful activities across the Division in 2024, creating opportunities for staff to actively participate in their own wellbeing journey, adapt to challenges, and thrive amidst a transforming workplace environment. The focus on mental and emotional wellness through both Division and Departmental initiatives served to enhance their ability to remain motivated and strong.

Regular Joyful Engagement meetings for Joy Ambassadors provide a platform for idea exchange and sharing of successful events, fostering mutual learning and ensuring a consistent flow of creative initiatives. Through this network, Joy Ambassadors serve as key points of contact for activities they have previously organized, enabling peer-to-peer support and replication of best practices across departments.

To ensure inclusivity and maximize resource utilization, smaller departments are encouraged to collaborate on joint activities, leveraging shared resources and building stronger interdepartmental connections. This collaborative approach ensures that all staff, regardless of department size, have access to impactful and joyful experiences. The flexibility of allowing staff to participate in activities of their choice further deepened engagement, as some stayed back to join these sessions, reinforcing camaraderie and collaborative spirit.

#### 3.4.2. Strengthening Leadership for Psychologically Safe Environments

Leadership development formed a cornerstone of engagement efforts through 25 “What Matters to You” (WMTY) conversations conducted across five Allied Health departments. These structured dialogs created psychologically safe spaces where staff could voice concerns, resulting in 34 actionable issues being documented and addressed. These issues ranged from identified challenges with work processes and workload, work-related stressors, concerns about career development as well as team dynamics. When appropriate, the WMTY groups provided a platform for participants to take ownership of identified issues and propose solutions. For example, one allied health department facing the increased patient flow discussed ways to optimize efficiency, including possibly attending to patients in batches. Another department discussed taking ownership of identifying relevant courses and speaking with management about career progression. For items that could not feasibly be addressed in the WMTY group itself, these were documented and shared with relevant stakeholders so that these needs could be properly addressed. For example, one department raised the possibility of increasing the flexibility of working arrangements. This was conveyed to the head of the department and was subsequently discussed more thoroughly in that department’s team meetings. The department then was able to implement work-from-home policies that allowed work arrangement flexibility whilst ensuring the department’s work operations were prioritized. It should also be noted that in this example, the individuals that raised this were relatively new to their department and had not raised this concern in previous department meetings. The WMTY group thus provided an opportunity whereby such needs could be communicated instead of being unheard.

This approach fostered open communication channels between leadership and staff, demonstrating commitment to inclusive team environments where every voice matters. The What Matters To You Conversation initiatives played a pivotal role in creating a workplace environment where staff felt heard, valued, and energized.

### 3.5. Ethical Considerations

As routine operational data collection annually for quality improvement of the initiatives implemented, the data from the survey was also de-identifiable, and approval from the institution research office was obtained to exempt from IRB review.

## 4. Results

### 4.1. Quantitative Findings

In total, 314 out of 715 allied health professionals (approximately 44% response rate) from Changi General Hospital completed the survey. The mean duration of employment at CGH was 8.89 years (standard deviation of 7.16) based on 307 valid responses (refers to Table 1). Participants were distributed across experience levels as follows: ≤5 years (41.7%), 6–10 years (25.1%), 11–15 years (16.9%), and >15 years (16.3%)

Table 2 includes the mean scores and standard deviations across the 13 aspects of workplace experience that contribute to staff wellbeing and engagement assessed in the survey. Happiness at work had a mean score of 2.86 (SD = 0.62), with 76.8% respondents indicating they were happy or very happy at work. The higher mean scores were for the aspects of meaningfulness at work (M = 3.33, SD = 0.60) with 95% agreeing or strongly agreeing that work was meaningful, job utilizing one’s skills (M = 3.18, SD = 0.59) with 92.4% agreeing or strongly agreeing as such, and being connected with co-workers (M = 3.02, SD = 0.69) with 82.8% agreeing or strongly agreeing as such. In contrast, lower mean scores were found for the aspects of being recognized by the organization (M = 2.58, SD = 0.80) with 40.8% disagreeing or strongly disagreeing that they felt recognized, as well as with respect to higher management being responsive and transparent (M = 2.66, SD = 0.81; M = 2.71, SD = 0.79, respectively), with 34.4% and 30.9% disagreeing or strongly disagreeing they experienced this.

### 4.2. Qualitative Findings

There were 28 qualitative responses to the question asking respondents to elaborate on their rating on how comfortable they were in sharing their opinions and concerns. Thematic analysis of these responses suggested several key themes reflecting the concerns of the staff. These were (1) lack of psychological safety, (2) lack of responsiveness and transparency by higher management, (3) unfair treatment by management as well as lack of adequate recognition.

### 4.3. Lack of Psychological Safety

A substantial proportion (50% of responses) indicated a key concern of the lack of psychological safety in sharing their concerns with management. For example, being fearful about being discriminated against, being asked to take on the extra workload of designing solutions or having concerns dismissed or explained away. Some representative quotes include “If we voice out, we’ll become the subject of unspoken targeting and blacklisting by the higher management“. “Who will dare to speak up to compromise their chances of getting work opportunities/promotion?”, “I (am) scared that giving feedback would mean that I will need to be coming out with solutions or extra workload that I could not handle.”, “After sharing opinions, it will always be shot down and justified with reasons from management that dismisses our concerns.”, “we are still afraid to make our concerns known especially since our interaction with higher management is only during townhall and people feel uncomfortable asking questions in public.”

### 4.4. Unresponsiveness and Lack of Transparency by Higher Management

There were quotes consistent with the quantitative findings that a substantial proportion of respondents did not find higher management responsive to needs, or transparent in communication. For example, “Sometimes feedback to higher management feels like creating more work for ourselves. Or we are encouraged to take a perspective change rather than addressing the main issue “, “Higher management has been giving predictable answers and make us feel that they just want to find ways to make us feel better but they aren’t tackling the root causes.”, “Inconsistencies in higher management. Quality rounds are conducted but there are often no real resolutions to the issues.”

### 4.5. Unfair Treatment and Lack of Adequate Recognition

Other qualitative responses also suggested the experience of unfair treatment by management (including work distribution), as well as the lack of adequate recognition. For example, “Unfair distribution of work, staff who are more vocal about their unhappiness get coddled “, “The management never values your hard work. If you don’t engage in extracurricular activities outside of clinical work, you won’t get promoted.”

As to the qualitative responses to the question on how staff would like to be recognized for their work, thematic analysis suggested the following key themes.

### 4.6. Better Monetary and Progression Rewards

Responses suggested a consistent wish for better monetary and progression rewards. For example, ‘’Please revise salary for AHP, seriously think that AHP is underpaid”, “Relooking at salary increment/adjustment for those that have worked for more than 10 years as starting salary for us is very low compared to the starting salary now”, “Promotion and salary raise. Some salaries are not meeting the market.”, “Faster promotion please, which depends on work performance”. Such quotes reflect issues pertaining to pay equity as well as career progression.

### 4.7. Recognition Gap

Another key theme in participants’ responses was that of the experience of a recognition gap between AHPs and other healthcare professionals. For example, ‘I believe the AHP staff are quite underpaid compared to doctors and nurses. Also, the recognition for the work performed by the AHP staff during COVID was not really celebrated as much as the doctors/nurses.”, and the desire of “Being respected as AHPs by other professions”.

## 5. Discussion

The cross-sectional survey of 314 AHPs at Changi General Hospital revealed a complex picture of workplace wellness and engagement characterized by a high endorsement of job happiness and meaningfulness but comparatively lower endorsement of organizational recognition, psychological safety, and higher management responsiveness and communication. These findings are early in the implementation of the staff wellbeing and engagement initiatives, but the survey does show positive outcomes as well as gaps and directions the Team can continue to work on.

### 5.1. Relating to Psychological Safety

The qualitative feedback regarding fear of retaliation, blacklisting and concerns being dismissed suggested that some AHPs seem to experience an environment in their teams where their voices as employees are suppressed. This raises concerns to address, given the evidence that psychological safety is essential for quality improvement, error reporting, and organizational learning in healthcare [21,22]. Studies including specific to allied health such as physiotherapists reveal hierarchical structures and interprofessional power dynamics frequently inhibit open communication [23,24]. Nembhard and Edmondson (2006) found that status differences between professions significantly impact psychological safety, with allied health professionals often experiencing lower levels compared to physicians [25]. Allied health professionals reported reluctance to voice concerns about patient care when it involved challenging medical decisions, despite their specialized expertise. When staff fear negative consequences for speaking up, patient safety may be compromised, as near-misses and system failures go unreported. The comment “if we voice out, we’ll become the subject of unspoken targeting and blacklisting” indicates a culture of silence in some teams that requires urgent attention. A review by Morrison (2014) demonstrates that employee silence is not merely the absence of voice, but an active withholding of information driven by fear of negative consequences [26]. In healthcare organizations, this silence can perpetuate inefficiencies, prevent innovation, and contribute to staff burnout.

The findings highlight the importance of establishing structured confidential feedback mechanisms (e.g., anonymous suggestion systems, third-party facilitated focus groups, regular pulse surveys) which will allow staff to raise concerns without fear of being identified or face retaliation. Additionally, findings also suggest that future interventions should facilitate managers to be trained in creating psychologically safe environments and responding constructively to feedback. Additionally, efforts should be made to establish clear anti-retaliation policies and accountability mechanisms. At the organizational level, the leaders should develop a systematic learning road map to create structural changes and implement core programs to encourage staff to speak up and report errors and near misses and to take proactive action when they notice potential for harm.

### 5.2. Relating to Rewards and Acknowledgement

Monetary rewards and the form of desired recognition reflecting issues about pay equity and competitive remuneration were raised. Whilst monetary compensation was the most frequently cited preference, the Team took the view that the desire for recognition extended beyond base salary alone. This has been echoed in the qualitative feedback which revealed that AHPs felt their COVID-19 contributions were “not really celebrated as much as the doctors/nurses” highlighting a broader recognition gap. AHPs often work behind the scenes, with their contributions less visible to patients, families, and the public compared to doctors and nurses. The literature reveals significant gaps in reward and acknowledgment systems for allied health professionals. Traditional healthcare reward structures have historically focused on medical and nursing achievements, with allied health contributions often overlooked. Research by Crozier et al. (2025) demonstrated that allied health professionals consistently report feeling invisible in organizational recognition programs [27]. This invisibility extends to organizational recognition systems, which may inadvertently prioritize more visible professions. This professional hierarchy, embedded in healthcare culture and organizational structures, perpetuates inequitable recognition and contributes to allied health workforce attrition [28].

Peer recognition programs were particularly effective for allied health professionals, who valued acknowledgment from colleagues who understood their professional challenges. Career advancement opportunities can also serve as a form of recognition. Research has shown that limited promotion pathways for allied health professionals contributed to feelings of professional stagnation [29].

All the evidence consistently demonstrates that psychological safety, fair treatment, and appropriate recognition are interconnected factors that significantly impact allied health professional satisfaction, retention, and ultimately patient care quality. Organizations that address these factors systematically show improved outcomes across multiple measures of healthcare team effectiveness [30,31,32].

The challenge remains for the Team’s Staff Profiling and Recognition arm to advocate for and help to implement a multi-tiered recognition program that includes both monetary (e.g., salary review, equity Adjustment, bonuses and performance incentives) and non-monetary (awards, public acknowledgement, career development opportunities) [33,34].

### 5.3. Management Responsiveness and Engagement

Concerns were also expressed in both the quantitative and qualitative survey findings on the lack of higher management transparency and responsiveness. The vital role of leadership in shaping staff wellbeing and engagement deserves more attention.

The comment that “interaction with higher management is only during townhall…” highlights structural barriers to communication. Townhalls, whilst valuable for information dissemination, are often ineffective for genuine dialog, with employees experiencing that it might be unsafe to openly communicate and ask questions [35]. The suggestion that “there should be other channels where staff can give suggestions” indicates a need for multiple, accessible feedback mechanisms that allow for confidential input.

The WMTY conversations were conducted by the department Joy Ambassadors who were not the department senior leadership members. While this encouraged openness among the participants and provided a safe platform for voicing concerns, there was no department leader present—a missed opportunity to clarify and address issues (e.g., disparity of expectations between management and staff). Potentially the absence of the leaders or Head of Department in these conversations also cast doubt if the concerns articulated will be further investigated. These findings further highlight the importance for hospitals’ higher management to develop genuine mechanisms for staff participation in decision-making, particularly on issues that directly affect their work. Future efforts to address education and leadership development programs designed to build agility, resilience, and compassionate leadership are needed. By equipping leaders to visibly support and champion wellness initiatives, it will help to embed a culture of psychological safety and wellbeing that permeates all levels of the organization. At the organization level, transformative shifts from a hierarchical, profession-centric culture to a collaborative, team-based culture that values all healthcare professions equitably are needed. This is to move beyond consultative approaches (where staff input is solicited but may be ignored) to participatory approaches (where staff have real influence on decisions). This requires sustained leadership commitment, role modeling from senior leaders, and embedding equity principles in all organizational policies and practices.

### 5.4. Joy at Work Framework

Overall, the adoption of the Joy at Work Framework shows how a structured, evidence-based approach can drive improvement in staff wellbeing and outcomes. In particular, the frontline initiatives to empower and have staff take ownership have worked well as seen in the achievement in the key areas of workplace experience, namely happiness at work and finding meaningfulness in their work. However, systemic cultural challenges persist, particularly around leadership accountability, equitable recognition across different allied health professions and creating a psychologically safe environment.

## 6. Limitation and Critique

AHPs can attend programs at cluster, institutional, division and department levels. The findings from the survey cannot be attributable to any specific but collective efforts. Like many at division and department levels in the Singhealth cluster, the Team may have conducted as many activities as deemed consistent to the overarching framework. Given that the AHD professional groups are very diverse and have different staff strength, it behooves the Team to be more cognizant of the corresponding challenges. It has to be very intentional and deliberate of the objectives, target audience, measures and sustainability of each of the initiatives and where each of these initiatives sit within the division, institutional and cluster framework. The challenge is determining and differentiating the impact if it is achieved at the individual program or through multiple participations or whether through the departmental, division, institutional or cluster-level intervention.

This survey was not originally statistically designed for research purposes. As noted above, it was developed by the team to track staff wellbeing and engagement. While relevant to the context it was designed for, the survey has not been statistically validated, which ultimately limits its statistical inference and the generalization of the findings. The Team will look into longitudinal studies to track satisfaction trajectories over time to identify critical inflection points where satisfaction declines and potential protective factors emerge. A refinement of the current survey tools will be needed.

The self-report measures are subject to response bias, including social desirability bias (though the high proportion of critical feedback suggests this was minimal) and recall bias. The use of anonymous surveys mitigates some of these concerns but cannot eliminate them entirely.

Whilst the qualitative data provided valuable insights, the open-ended responses were brief and not systematically probed. In-depth interviews or focus groups would provide richer understanding of the mechanisms underlying dissatisfaction and potential solutions.

## 7. Conclusions

In 2023, the biennial SingHealth Employee Engagement survey results pointed to the AHD three areas of concern related to human resources and work–life balance, recognition and appreciation of work and innovation. The 2024 SingHealth Staff Wellness Pulse Survey conducted for the first time showed that the Division demonstrated high levels of reported mental wellbeing, resilience, and psychological safety. In the 2024 CGH Employee Engagement Survey, the Division showed steady improvement in the scores for mental health, work–life, career development and organization culture compared to the surveys conducted in 2021 and 2023. The Division’s staff attrition rate has also been falling from 13.9% in 2022, 10.7% in 2023, 8.9% in 2024 and, to date, 6.7% in October 2025. This paper attempts to look at how one Division within a healthcare institution advances its staff wellbeing and engagement. It demonstrates that it requires a sustained, integrated approach that empowers individuals, strengthens leadership, and solidifies institutional commitment. In fact, on the latter, the cluster and hospital play a vital role to continue through infrastructure and policy enhancement to support and reinforce staff wellbeing and engagement. The Division will further benefit from the staff wellness portal which SingHealth will launch to serve as a comprehensive resource hub and the strengthening of peer support networks. The platform will facilitate access to information, raise awareness, and promote open dialog about mental health challenges, embedding wellness as a core organizational value. Overall, the findings support a comprehensive, system-level commitment to staff wellbeing for a thriving healthcare workforce capable of meeting the evolving demands of healthcare delivery while maintaining compassionate, high-quality patient care.

## Figures and Tables

**Table 1:** Participant demographics.

Years of Experience at CGH	n	%
≤5 years	128	41.7
6–10 years	77	25.1
11–15 years	52	16.9
>15 years	50	16.3
Mean (SD)	8.89 (7.16)	

**Table 2:** Aspects of workplace experience contributions to staff wellbeing and engagement.

Aspects of Workplace Experiences	Mean (And Standard Deviation)	Rating Distribution			
		1	2	3	4
Happiness at work	2.86 (0.62)	1.9%	21.3%	65.3%	11.5%
Job utilizes one’s skills and abilities	3.18 (0.59)	1.3%	6.4%	65.6%	26.8%
Meaningfulness of work	3.33 (0.60)	1%	4.1%	56.1%	38.9%
Work is distributed evenly	2.78 (0.76)	6.1%	23.6%	56.4%	14%
Being valued for one’s contributions	2.86 (0.78)	6.4%	19.1%	57%	17.5%
Being connected to co-workers	3.02 (0.69)	2.9%	14.3%	61.1%	21.7%
Being recognized by department	2.82 (0.79)	8.3%	16.9%	59.2%	15.6%
Being recognized by organization	2.58 (0.80)	10.5%	30.3%	49.7%	9.6%
Manager advocates for one’s needs	2.88 (0.77)	7.6%	13.1%	62.7%	16.6%
Higher management is responsive to one’s needs	2.66 (0.81)	10.5%	23.9%	54.5%	11.1%
Higher management is transparent and clear in communication	2.71 (0.79)	9.2%	21.7%	57.6%	11.5%
Trust and rapport with manager	2.98 (0.73)	5.4%	11.8%	62.4%	20.4%
Comfortable sharing opinions with management	2.79 (0.80)	9.2%	17.2%	58.9%	14.6%

Scale: 1 = strongly disagree/very unhappy, 2 = disagree/unhappy, 3 = agree/happy, 4 = strongly agree/very happy.

## Data Availability

Dataset available on request from the authors. The raw data supporting the conclusions of this article will be made available by the authors on request. The release of the data is subjected to approval from the institution which owns the data.

## Abbreviations

The following abbreviations are used in this manuscript:

CGH	Changi General Hospital
MOM	Ministry of Manpower
EAP	Employee Assistance Program
WMTY	What Matters to You
AHD	Allied Health Division
AHP	Allied Health Professional
